# Greater Symptom Burden and Poorer Quality of Life Outcomes Are Associated With The Co-Occurrence of Anxiety and Depression During Cancer Chemotherapy

**DOI:** 10.1016/j.soncn.2025.151809

**Published:** 2025-02-13

**Authors:** Alejandra Calvo-Schimmel, Marilyn J. Hammer, Yvette P. Conley, Steven M. Paul, Bruce A. Cooper, Joosun Shin, Carolyn Harris, Lisa Morse, Jon D. Levine, Christine Miaskowski

**Affiliations:** a American Cancer Society, Atlanta, GA; b Dana Farber Cancer Institute, Boston, MA; c School of Nursing, University of Pittsburgh, Pittsburgh, PA; d School of Nursing, University of California, San Francisco, CA; e School of Medicine, University of California, San Francisco, CA

**Keywords:** Anxiety, Cancer, Chemotherapy, Depression, Latent profile analysis, Quality of life

## Abstract

**Objectives:**

Anxiety and depression are common symptoms in oncology patients undergoing chemotherapy. Study purpose was to evaluate for differences in severity of common symptoms (ie, fatigue, energy, sleep disturbance, cognitive function, pain) and quality of life (QOL) outcomes among three subgroups of oncology outpatients with distinct joint anxiety and depression profiles.

**Methods:**

Oncology outpatients (N = 1328) completed measures of state anxiety and depression, six times over two cycles of chemotherapy. Latent profile analysis was done to identify subgroups of patients with distinct joint state anxiety AND depression profiles. Patients completed measures of trait anxiety, morning and evening fatigue, morning and evening energy, sleep disturbance, cognitive function, and pain, as well as generic and disease-specific measures of QOL at enrollment. Differences among the classes in symptom severity scores and QOL scores were evaluated using parametric and non-parametric tests.

**Results:**

Three distinct joint anxiety AND depression profiles were identified and named: Low Anxiety and Low Depression (57.5%, Both Low), Moderate Anxiety and Moderate Depression (33.7%, Both Moderate), and High Anxiety and High Depression (8.8%, Both High). All of the symptom severity scores showed a “dose-response effect” (ie, as the joint anxiety AND depression profiles worsened, the severity of all of the symptoms increased). Likewise, for both the general and disease-specific QOL (except spiritual well-being) measures, all of the scores decreased as the joint anxiety AND depression profiles worsened. Compared to the Both Low classes, the other two classes reported lower scores for the spiritual well-being domain.

**Conclusions:**

More than 40% of patients receiving chemotherapy experience moderate to high levels of both anxiety AND depression. These patients report an extremely high symptom burden and significant decrements in all domains of QOL.

**Implications for Nursing Practice:**

Clinicians need to perform comprehensive assessments of depression and anxiety and other common symptoms and QOL outcomes during chemotherapy. In addition, referrals for targeted interventions are needed to manage multiple symptoms and improve patients’ QOL.

Anxiety and depression are two of the most common symptoms experienced by oncology patients undergoing chemotherapy.^1^ Unrelieved anxiety and depression, as individual symptoms, are often associated with increases in symptom burden,^2,3^ longer hospitalizations,^2^ financial toxicity,^2^ decrements in quality of life (QOL),^2,4^ and poorer functional^5^ and survival^4,6^ outcomes. However, limited evidence from our group^7^ and others^8–10^ suggests that anxiety and depression often co-occur among patients with cancer and that they are moderately correlated.^11^ While combined state anxiety and depressive symptoms (CADS) are reported by 12.4%^9^ to 28.0%^10^ of oncology patients, to our knowledge, only two longitudinal studies by our team^7,12^ examined the occurrence, severity, and associated risk factors for CADS in these patients.

In the first 6-month longitudinal study that combined data from growth mixture modeling analyses of anxiety^13^ and depression^14^ as single symptoms and evaluated for differences in demographic and clinical characteristics and QOL in women before breast cancer surgery,^7^ four distinct anxiety AND depressive symptom groups were identified (ie, Lower Anxiety and Resilient [32.5%]; Lower Anxiety and Subsyndromal Depressive symptoms [9.3%]; Higher Anxiety and Resilient [11.6%]; Higher Anxiety and Subsyndromal Depressive symptoms [44.5%]). Risk factors associated with membership in the Higher Anxiety and Subsyndromal Depressive symptoms class included being younger, self-reporting a non-White ethnicity, having a poorer functional status, receiving neoadjuvant or adjuvant chemotherapy, having greater difficulty dealing with the disease and its treatment(s), and reporting less social support. In addition, women in this class had a poorer QOL.

In the second 2-month longitudinal study of patients with heterogeneous types of cancer that used latent profile analysis (LPA),^12^ three classes of patients with distinct joint state anxiety AND depression profiles were identified (ie, Low Anxiety and Low Depression [57.5%]; Moderate Anxiety and Moderate Depression [33.7%], High Anxiety and High Depression [8.8%]). For the two worst profiles, younger age, self-reporting a Hispanic or mixed ethnicity, living alone, being unmarried, being unemployed, having a lower annual income, having a poorer functional status, and having a higher comorbidity burden were the identified risk factors. In addition, patients in the two worst classes reported higher levels of global and cancer-specific stress; higher occurrence rates and effects from previous stressful life events; lower levels of resilience; and a higher use of disengagement coping strategies (eg, substance use).

While these two longitudinal studies provide useful information on the co-occurrence of anxiety and depression and associated risk factors, only one study^12^ evaluated patients receiving chemotherapy. In addition, only one study^7^ examined the relationships between the co-occurrence of anxiety and depression and QOL outcomes. Of note, neither of these studies^7,12^ determined if patients who had higher levels of both anxiety and depression experienced a higher overall symptom burden. Therefore, the current study evaluates for differences in the severity of common symptoms associated with cancer and its treatments (ie, sleep disturbance, fatigue, decrements in energy, cognitive dysfunction, and pain) and QOL outcomes among three previously identified groups of patients with distinct joint state anxiety AND depression profiles.^12^ We hypothesize that patients in the two worst joint state anxiety AND depression profiles will report higher levels of all of the symptoms evaluated and poorer QOL outcomes.

## METHODS

### Patients and Settings

The theoretical framework for the parent study was the Theory of Symptom Management.^15^ Specifically, this study focused on the symptom and outcome (ie, QOL) domains. This study is part of a larger, longitudinal study of the symptom experience of oncology outpatients receiving chemotherapy.^16^ Briefly, patients were ≥18 years of age; had a diagnosis of breast, gastrointestinal, gynecological, or lung cancer; had received chemotherapy within the preceding 4 weeks; were scheduled to receive at least two additional cycles of chemotherapy; were able to read, write, and understand English; and provided written informed consent. Patients were recruited from two comprehensive cancer centers, one Veterans Affairs hospital, and four community-based oncology programs during their first or second cycle of chemotherapy.

### Study Procedures

The study was approved by the institutional review board at each of the study sites. Of the 2234 patients approached, 1343 provided written informed consent to participate. The major reason for refusal was being overwhelmed by their cancer treatments. Eligible patients were approached by a member of the research team in the infusion unit to discuss participation in the study during their first or second cycle of chemotherapy.

Patients completed the Spielberger State Anxiety Inventory (STAI-S)^17^ and Center for Epidemiologic Studies Depression Scale (CES-D),^18^ in their homes, a total of six times over two chemotherapy cycles (ie, before chemotherapy administration, approximately 1 week after chemotherapy administration, and approximately 2 weeks after chemotherapy administration). All of the other measures were assessed at enrollment (ie, before the second or third cycle of chemotherapy). Medical records were reviewed for disease and treatment information. For this analysis, data from patients who had complete data on the anxiety and depression measures (N = 1328) were evaluated.

### Instruments

#### Demographic and clinical measures

Patients completed a demographic questionnaire, Karnofsky Performance Status (KPS) scale,^19^ Self-Administered Comorbidity Questionnaire (SCQ),^20^ Alcohol Use Disorders Identification Test (AUDIT),^21^ and a smoking history questionnaire. The toxicity of each patient’s chemotherapy regimen was rated using the MAX2 score.^22^ Medical records were reviewed for disease and treatment information.

#### Anxiety and depression measures

The Spielberger State-Trait Anxiety Inventories (ie, STAI-T and STAI-S) were used to evaluate anxiety.^17^ The STAI-S measures a person’s temporary anxiety response to a specific situation or how anxious or tense a person is “right now” in a specific situation. The STAI-T measures a person’s predisposition to anxiety as part of one’s personality. Cutoff scores of ≥31.8 or ≥32.2 indicate high levels of trait and state anxiety, respectively. Cronbach’s alphas for the STAI-T and STAI-S were .92 and .96, respectively.

The 20-item CES-D evaluates the major symptoms in the clinical syndrome of depression.^18^ A total score can range from 0 to 60, with scores of ≥16 indicating the need for individuals to seek clinical evaluation for depression.^23^ The CES-D has four subscale scores that measure different symptom domains within depression (ie, somatic symptoms, depressed affect, positive affect, interpersonal problems). Cronbach’s alpha for the CES-D total score was .89.

#### Other common symptom measures

The 21-item General Sleep Disturbance Scale (GSDS) was designed to assess various aspects of sleep disturbance (ie, quality, quantity, onset latency, mid and early awakenings, sleep medications, daytime sleepiness). Each item was rated on a 0 (never) to 7 (everyday) numeric rating scale (NRS). The GSDS total score ranges from 0 (no disturbance) to 147 (extreme sleep disturbance). Each mean subscale score ranges from 0 to 7.^24–26^ Subscale scores of ≥3 and a GSDS total score of ≥43 indicate a significant level of sleep disturbance that warrants clinical evaluation and management.^27^ Cronbach’s alpha for the GSDS total score was .83.

The 18-item Lee Fatigue Scale (LFS) was designed to assess physical fatigue and energy.^28^ Each item was rated on a 0 to 10 NRS. Total fatigue and energy scores were calculated as the mean of the 13 fatigue items and the 5 energy items, respectively. Higher scores indicate greater fatigue severity and higher levels of energy.

Using separate LFS questionnaires, patients were asked to rate each item based on how they felt within 30 minutes of awakening (ie, morning fatigue, morning energy) and prior to going to bed (ie, evening fatigue, evening energy). The LFS has established cut-off scores for clinically meaningful levels of fatigue (ie, ≥3.2 for morning fatigue, ≥5.6 for evening fatigue) and energy (ie, ≤6.2 for morning energy, ≤3.5 for evening energy).^27^ Cronbach’s alphas were 0.96 for morning and 0.93 for evening fatigue and 0.95 for morning and 0.93 for evening energy.

The 16-item Attentional Function Index (AFI) was designed to measure attentional function.^29^ A higher total mean score on a 0 to 10 NRS indicates greater capacity to direct attention.^29^ Total scores are grouped into categories of attentional function (ie, <5.0 low function, 5.0 to 7.5 moderate function, >7.5 high function).^30^ In addition, the AFI has three subscales (ie, effective action, attentional lapses, interpersonal effectiveness). Cronbach’s alpha for the total AFI score was 0.93.

Worst pain severity was assessed using the Brief Pain Inventory (BPI).^31^ Patients were asked to indicate whether they were generally bothered by pain (yes/no). If they were generally bothered by pain, they indicated if they had non-cancer pain, cancer pain, or both types of pain. Then, patients rated their worst pain severity in the past 24 hours using a 0 (no pain) to 10 (worst pain imaginable) NRS. For the pain interference items, a total mean score was calculated.

#### QOL measures

QOL was evaluated using generic (ie, Medical Outcomes Study-Short Form-12 [SF-12]^32^) and disease-specific (ie, Multidimensional QOL Scale-Patient Version [MQOLS-PV]^33^) measures. The individual items on the SF-12 were evaluated and the instrument was scored into two component scores (ie, physical component summary [PCS] and mental component summary [MCS]). MQOLS-PV measures four domains of QOL (ie, physical, psychological, social, and spiritual well-being), as well as a total QOL score. For both measures, higher scores indicate a better QOL.

#### Data Analysis

As described previously,^12^ LPA was used to identify unobserved subgroups of patients (ie, latent classes) with distinct joint anxiety AND depression profiles over the six assessments using the patients’ STAI-S and CES-D scores. In brief, LPA was performed using Mplus version 8.4.^34^ Estimation was carried out with full information maximum likelihood with standard errors and a chi-squared test that is robust to non-normality and non-independence of observations (“estimator=MLR”). Model fit was evaluated to identify the solution that best characterized the unobserved latent class structure with the Bayesian Information Criterion,^35^ Vuong-Lo-Mendell-Rubin likelihood ratio test, entropy, and latent class percentages that were large enough to be reliable.^34,36^ Missing data were accommodated for with the use of the Expectation-Maximization algorithm.^37^

Data were analyzed using IBM SPSS Statistics version 29 (IBM Corporation, Armonk, NY). Differences among the joint anxiety AND depression latent classes in symptom severity scores and QOL outcomes at enrollment were evaluated using parametric and nonparametric tests. A Bonferroni corrected *P* value of <.017 was considered statistically significant for the pairwise contrasts (ie, 0.05/3 possible pairwise contrasts).

## RESULTS

### Latent Profile Analysis

As described previously,^12^ three latent classes were identified and named Low Anxiety and Low Depression (57.5%, Both Low); Moderate Anxiety and Moderate Depression (33.7%, Both Moderate); and High Anxiety and High Depression (8.8%, Both High) based on the clinically meaningful cutoff scores for the STAI-S^17^ and CES-D.^18^ For the Both Low and Both Moderate classes, while anxiety scores remained relatively stable across the two cycles of chemotherapy, depression scores increased slightly at the second and fifth assessments (ie, following the administration of chemotherapy). For the Both High class, while anxiety scores decreased at the second assessment and increased at each of the subsequent assessments, depression scores remained relatively stable (Supplemental Fig 1).

### Sample Characteristics

In brief, the overall sample (N = 1328) was predominantly female, White, and college educated. In terms of differences in demographic and clinical characteristics among the three latent classes,^12^ compared to Both Low class, the other two classes were younger; were more likely to self-report being of Hispanic or mixed ethnicity; were more likely to live alone; and were less likely to be employed (Supplemental Table 1). In addition, significant differences were found among the three latent classes in marital status and KPS scores (ie, Both Low > Both Moderate > Both High), as well as annual household income, total number of comorbid conditions, SCQ scores, and self-reported diagnoses of depression and back pain (ie, Both Low < Both Moderate < Both High).

### Common Symptoms

As shown in Table 1, significant differences were found among the three latent classes in trait anxiety, morning and evening fatigue, worst pain intensity, and pain interference scores (ie, Both Low < Both Moderate < Both High), as well as in morning and evening energy scores (ie, Both Low > Both Moderate > Both High). In addition, significant differences were found among the three latent classes in the occurrence of both cancer and non-cancer pain (ie, Both Low < Both Moderate < Both High).

Table 2 summarizes the differences among the three latent classes in the total, as well as each subscale scores for the CES-D, GSDS, and AFI.

### Depressive Symptoms

As shown in Table 2, significant differences were found among the three latent classes for the somatic symptoms, depressed affect, and interpersonal problems subscale and total CES-D scores (ie, Both Low < Both Moderate < Both High), as well as for the positive affect subscale score (ie, Both Low > Both Moderate > Both High).

### Sleep Disturbance

Significant differences were found among the three latent classes for the sleep quality, sleep onset latency, early awakenings, use of sleep medications, and excessive daytime sleepiness subscale scores, as well as for the total GSDS score (ie, Both Low < Both Moderate < Both High); Table 2). Compared with the Both Low class, the Both High class reported higher scores for sleep quantity (ie, fewer hours of sleep) and midsleep awakenings.

### Cognitive Function

Significant differences were found among the three latent classes for the effective action, attentional lapses, and interpersonal effectiveness subscale scores, as well as for the total AFI score (ie, Both Low > Both Moderate > Both High; Table 2).

### Differences in QOL Outcomes

**Fig 1.**
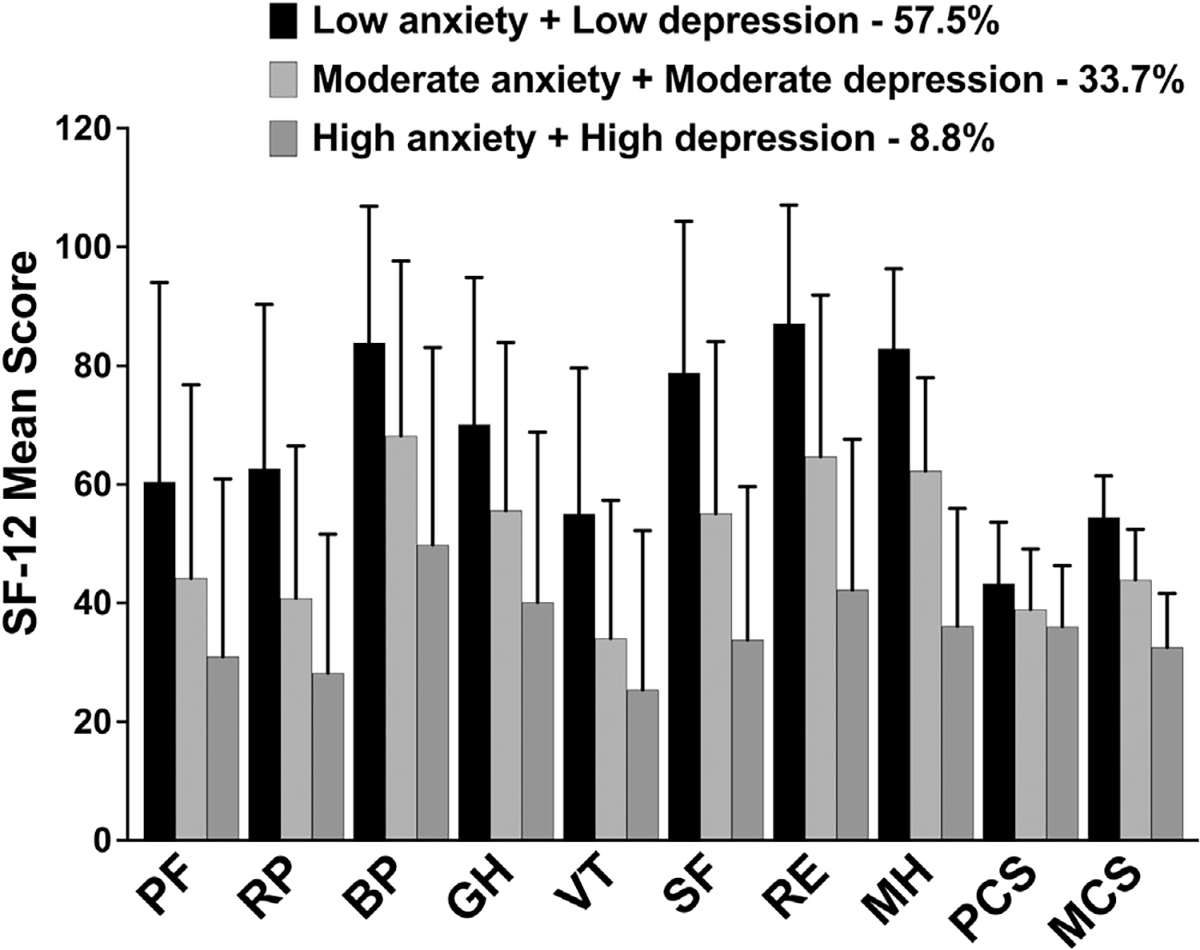
Differences in Medical Outcomes Study-Short Form 12 (SF-12) physical functioning (PF), role physical (RP), bodily pain (BP), general health (GH), vitality (VT), social functioning (SF), role emotional (RE), mental health (ME), physical component summary (PCS), and mental component summary (MCS) scores among the joint anxiety and depression latent classes. All values are plotted as means ± SDs. For all the SF-12 subscales, as well as the PCS and MCS scores, post hoc contrasts demonstrated significant differences among the classes that followed the same pattern (ie, low > moderate > high; all, *P* < .05).

**Fig 2.**
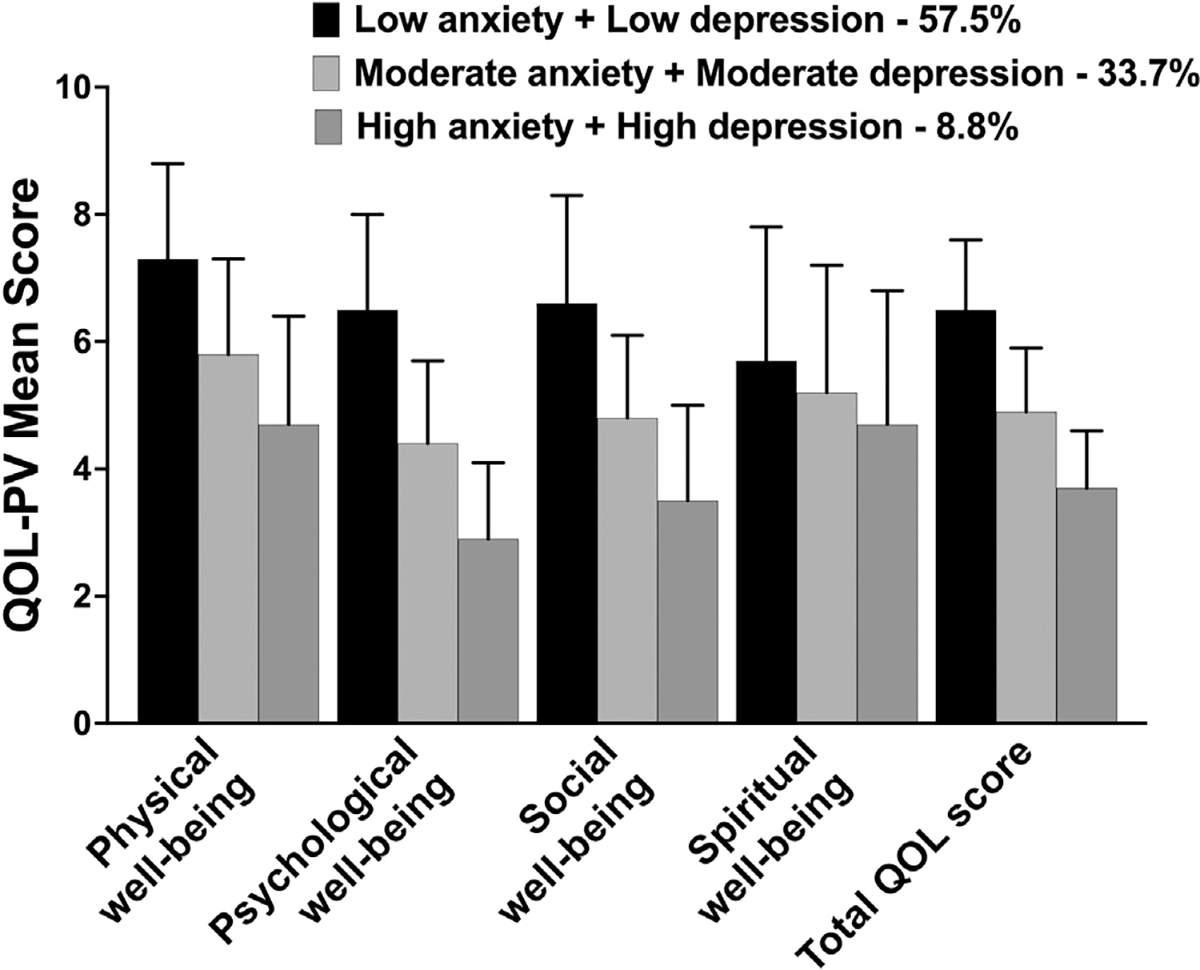
Differences in Quality-of-Life Scale—Patient Version (QOL-PV) scores for the physical, psychological, social, and spiritual well-being domains as well as total quality of life (QOL) among the joint anxiety and depression latent classes. All values are plotted as means ± SDs. For the physical, psychological, and social domains, as well as QOL total scores, post hoc contrasts demonstrated significant differences among the classes that followed the same pattern (ie, low > moderate > high; all, *P* < .05). For the spiritual well-being domain, the post hoc contrasts demonstrated that the differences among the classes were as follows: low > moderate and high classes (*P* < .05).

Significant differences were found among the three latent classes for all of the SF-12 subscales, as well as the PCS and MCS scores (ie, Both Low > Both Moderate > Both High; Fig 1). In addition, significant differences were found among the three latent classes for the MQOLS-PV physical, psychological, and social well-being scores, as well as for the total QOL scores (ie, Both Low > Both Moderate > Both High; Fig 2). For the spiritual well-being domain, compared to the Both Low class, the other two classes reported lower scores.

## DISCUSSION

This study is the first to evaluate for differences in common symptoms associated with cancer and its treatments and QOL outcomes among these classes. Consistent with prior studies that found associations between higher levels of anxiety and depression, as individual symptoms, and higher levels of other common cancer-related symptoms,^38–40^ as well as poorer QOL outcomes,^41^ our a priori hypothesis was supported in that patients with the two worst joint symptom profiles reported higher scores for all of the common symptoms evaluated and lower scores for both QOL measures. In fact, for all the common symptoms evaluated, severity scores demonstrated a “dose-response effect” in that, as the joint anxiety AND depression profiles worsened, all of the symptom scores changed (ie, either increased or decreased) in a stepwise fashion.

### Fatigue and Energy

For the Both Moderate and Both High classes, morning and evening fatigue scores were above the clinically meaningful cutoff scores. Likewise, for these two classes, their morning and evening energy scores represent clinically meaningful decrements in these symptoms. While evidence suggests that anxiety and depression are moderately to highly correlated^42–46^ and have bidirectional relationships with other symptoms (eg, fatigue,^40^ sleep disturbance^38^), our study is the first to describe a dose-response relationship between diurnal variations in both fatigue and energy levels and worsening of the joint anxiety and depression profiles in oncology patients receiving chemotherapy.

### Depressive Symptoms

More than 42.0% of our patients reported a CES-D total score that suggests clinically meaningful levels of depressive symptoms. Our prevalence rate is at the higher end of the 17% to 45% range reported in one systematic review.^47^ These differences in prevalence rates may be related to various measures used to evaluate depressive symptoms; how caseness was determined; the timing of the assessments; and/or sample characteristics. Of note, 26.3% and 60.3% of the patients in the Both Moderate and Both High classes, respectively, self-reported a diagnosis of depression on the SCQ.

For all of the CES-D subscale scores, the differences among the three latent classes demonstrated a dose-response effect. These differences represent not only statistically significant, but clinically meaningful differences (ie, somatic symptoms [*d* = 1.2 to 2.6]; depressed affect [*d* = 1.4 to 4.5]; positive affect [*d* = −1.0) to −2.3]; interpersonal problems [*d* = 0.3 to 1.6]).^48^ Our findings are consistent with a previous report that found that patients with advanced cancer who had higher scores for all four CES-D subscales reported higher state anxiety scores.^49^

As noted in a study that confirmed the four-factor structure of the CES-D,^50^ clinicians can use the CES-D subscale scores to assess for patterns of improvement or lack of improvement in various aspects of depression. These authors noted that patients may become less “blue” (ie, depressed affect) over time while becoming more “hopeless” (ie, positive affect) and more “fearful” (ie, interpersonal effectiveness). Changes in patients’ CES-D subscale scores could be used to develop tailored interventions to treat specific types of depressive symptoms. Future studies need to evaluate for changes over time in each of the subscales of the CES-D, as well as for joint anxiety and CES-D subscale profiles.

### Sleep Disturbance

It is important to note that, regardless of class membership, all of the patients in the current study had a total GSDS score above the clinically meaningful cutoff. Furthermore, the total GSDS scores for the Both Moderate and Both High classes are comparable to those reported by oncology patients undergoing other types of cancer treatment (eg, surgery [56.2],^51^ radiation therapy [45.4]^52^). This finding is consistent with a systematic review that noted the that bidirectional relationships exist among anxiety, depression, and sleep disturbance due to common inflammatory and neurotransmitter pathways.^38^

Compared to the Both Low class, the other two classes reported significant decrements in sleep quality, as well as problems with sleep initiation (ie, sleep onset latency), sleep maintenance (ie, early awakenings, midsleep awakenings), and excessive daytime sleepiness for greater than three days per week. Equally important, except for the quantity of sleep and mid-sleep awakenings, all of the other GSDS subscales scores demonstrated a “dose-response effect.” Our results are consistent with previous reports that found that sleep disturbance was a common problem in oncology patients who experienced anxiety and/or depression during treatment.^53,54^ One plausible explanation is that patients in the Both Moderate and Both High classes had a history of poor sleep before the initiation of chemotherapy.^55^ Equally plausible, while most anxiolytics improve sleep,^56^ some antidepressants can cause sleep disturbance.^57^ For example, fluoxetine and venlafaxine are associated with difficulties with sleep maintenance (ie, mid and early awakenings).^57^ Given that detailed information on the use of anxiolytics and antidepressants was not available, future studies need to collect these data and evaluate their impact on the co-occurrence of anxiety and depression, as well as sleep disturbance.

### Cognitive Function

In terms of cognitive function, significant differences were found among the three classes in all of the subscales and total AFI scores and these differences demonstrated a dose-response effect. Furthermore, compared to the Both Low class, the Both Moderate and Both High classes had total AFI scores that indicate moderate (5.6) and low (4.4) levels of cognitive function, respectively. Of note, 39.2% of the patients in the Both Moderate class had an AFI score that suggests low levels of cognition (ie, <5). Our findings are consistent with prior reports that suggest that an association exists between higher levels of anxiety^58,59^ and depression,^59,60^ as single symptoms, and lower levels of cognitive function during chemotherapy.

While specific clinically meaningful cutoff scores for the effective action, attentional lapses, and interpersonal effectiveness subscales are not available,^61^ if the cutoff scores for the total AFI (ie, <5.0 = Low, 5 to 7.5 = Moderate, >7.5 = High) are used, it is not surprising that the Both Moderate and Both High classes had mean scores, across all three subscales, that indicate moderate and low levels of cognitive function, respectively. In terms of the effective action subscale, which evaluates a patient’s ability to engage in purposeful actions (ie, reasoning, planning, executing, problem-solving),^61^ our findings suggest that these types of actions may be more challenging for patients with higher levels of anxiety and depression. For example, in a study that investigated attention and memory in newly diagnosed breast cancer patients compared to healthy controls,^62^ patients who experienced higher levels of psychological distress (ie, anxiety and/or depression) showed slower processing speed and poorer verbal memory. Decreases in processing speed, poorer verbal memory, and difficulties with sustained concentration (ie, attentional lapses) may diminish patients’ ability to reason, plan, and execute actions of daily life effectively,^63^ which in turn, may have a negative impact on QOL.^61^

In terms of interpersonal effectiveness, our findings suggest that the Both Moderate and Both High classes encounter moderate to severe difficulties maintaining meaningful personal relationships. One plausible explanation for this finding is the social isolation associated with being unemployed^59^ and/or decreases in social activities.^64^ Of note, 72.2% of the patients in the Both Moderate and 75.9% of the patients in the Both High classes were unemployed. In addition, lower levels of social well-being were reported by these two classes.

### Pain

Consistent with previous studies that found that oncology patients who experience anxiety and/or depression reported more severe pain and greater interference with daily activities,^46,65^ the Both Moderate and Both High classes had higher rates of both cancer and noncancer pain. In addition, 30.4% to 45.7% of the patients in these two classes reported the occurrence of back pain. Our findings are consistent with previous reports in oncology patients. For example, in a study that evaluated for associations among pain, anxiety, and depression,^66^ compared to pain-free controls, oncology patients with pain had significantly worse anxiety and depressed mood. In another study,^46^ compared to patients without pain, oncology patients with pain were 4.44 and 2.29 times more likely to report anxiety and depression, respectively. Furthermore, as the severity of pain increased, the prevalence of anxiety and depression increased by 23.6% and 19%, respectively.

### Potential Mechanisms

The co-occurrence of anxiety and depression and other common symptoms in oncology patients may be partially explained by the fact that these symptoms share common underlying mechanisms. For example, a growing body of evidence suggests that changes in inflammatory responses contribute to higher levels of anxiety,^67^ depression,^68,69^ fatigue,^70,71^ sleep disturbance,^72^ cognitive impairment,^73^ and pain.^74^ In addition, alterations in the hypothalamic-pituitary-adrenal axis that result in changes in neuronal excitability and synthesis and release of various neurotransmitters may be another shared mechanism. As noted in various reviews, neuroinflammation is associated with anxiety,^75^ depression,^75^ fatigue,^70,71^ sleep disturbance,^72,76^ cognitive impairment,^77^ and pain.^74,78,79^ A third potential common mechanism is changes in the gut-brain axis. The administration of chemotherapy is associated with intestinal inflammation and alterations in its permeability that results in changes in the gut microbiome. Evidence suggests that alterations in the gut microbiome contributes to anxiety,^80^ depression,^81,82^ fatigue, sleep disturbance,^76,83^ cognitive impairment,^84–87^ and pain.^88–90^ Future research will need to determine the relative contribution of each of these mechanisms to the occurrence and severity of each of the symptoms and to the overall symptom burden of patients with cancer.

Equally plausible, the type and duration of chemotherapy may play a role in the development and/or exacerbation of these common symptoms. For example, several lines of evidence suggest that certain types of chemotherapy (eg, anthracyclines) are associated with higher levels of anxiety,^91^ depression,^91^ sleep disturbance,^92^ fatigue,^93^ cognitive impairment,^94^ and pain.^95^ In addition, several studies found that a longer duration of chemotherapy treatment contributes to a progressively higher symptom burden.^94,96–100^

### QOL Outcomes

Consistent with prior studies that reported associations between anxiety^41,101^ and depression,^41,102^ as single symptoms, and decrements in QOL in oncology patients during chemotherapy, both the general and cancer-specific QOL (except spiritual well-being) outcomes demonstrated a “dose-response effect” (Figs 1 and 2). In addition, the differences across the various domains of QOL represent not only statistically significant but clinically meaningful decrements in QOL (*d* = 0.43 to 2.77).^48^ Of note, patients in Both Moderate and Both High classes reported PCS and MCS scores of less than 50, which are lower than the normative scores for the general population.^32^

Our findings can be partially explained by the fact that oncology patients often experience multiple co-occurring symptoms associated with chemotherapy that may result in additive or synergistic effects. In turn, these effects affect the physical, psychological, and social aspects of the patients’ daily life.^103^ For example, in one study,^104^ similar to our findings, oncology patients who experienced psychiatric comorbidity (ie, anxiety AND depression) had lower scores on all eight dimensions of the Short Form Health Survey-8 as well as for the PCS and MCS components.

### Limitations

Our study has some limitations. While large, our sample was relatively homogenous in terms of gender, race/ethnicity, and socioeconomic status, so our findings may not be representative of all oncology patients. In addition, because anxiety and depression were measured over only two cycles of chemotherapy, research is needed that evaluates for changes in these two symptoms from before through the completion of chemotherapy, as well as for changes in modifiable risk factors. Information on anxiolytics and antidepressants was not collected and may have assisted with the interpretation of the study’s findings. Lastly, the major reason for refusal to participate was being overwhelmed by cancer treatment, which may have led to underestimation of anxiety and depression in this sample.

### Conclusions and Implications for Clinical Practice

Despite these limitations, this study is the first to evaluate for differences in the severity of common symptoms and QOL outcomes in patients undergoing chemotherapy with distinct joint state anxiety and depression profiles. Given that clinically meaningful levels of anxiety and depression can persist following the completion of chemotherapy,^105^ clinicians need to conduct comprehensive assessments routinely for the co-occurrence of these two symptoms, as well as other common symptoms to identify high risk patients. In addition, clinicians need to initiate referrals for individualized interventions (eg, meditation, sleep management) that patients can use to manage multiple co-occurring symptoms

## Supplementary Material

MMC1

MMC3

MMC2

Supplementary materials

Supplementary material associated with this article can be found in the online version at doi:10.1016/j.soncn.2025.151809.

## Figures and Tables

**Table 1:** Differences in Symptom Severity Scores Among the Joint State Anxiety and Depression Latent Classes at Enrollment

Symptoms^a^	Low Anxiety and Low Depression (0) 57.5% (n = 764)	Moderate Anxiety and Moderate Depression (1) 33.7% (n = 448)	High Anxiety and High Depression (2) 8.8% (n = 116)	Statistics
	Mean (SD)	Mean (SD)	Mean (SD)	

Trait anxiety (≥31.8)	28.9 (5.8)	41.1 (7.6)	53.5 (8.8)	F=902.75, *P* < .001 0 < 1 < 2
Morning fatigue (≥3.2)	2.3 (1.9)	3.9 (2.1)	5.8 (2.0)	F=212.64, *P* < .001 0 < 1 < 2
Evening fatigue (≥5.6)	4.8 (2.1)	5.7 (1.9)	7.0 (1.8)	F=68.68, *P* < .001 0 < 1 < 2
Morning energy (≤6.2)	4.8 (2.3)	4.0 (2.0)	3.3 (2.0)	F=38.27, *P* < .001 0 > 1 > 2
Evening energy (≤3.5)	3.8 (2.1)	3.4 (1.9)	2.6 (2.1)	F=19.75, *P* < .001 0 > 1 > 2
	% (n)	% (n)	% (n)	
Types of pain				X^2^=94.63, *P* < .001
None	31.5 (265)	20.0 (87)	5.3 (6)	0 > 1 > 2
Only non-cancer pain	24.5 (185)	28.7 (125)	28.1 (32)	NS
Only cancer pain	17.5 (132)	14.3 (62)	11.4 (13)	NS
Both non-cancer and cancer pain	22.8 (172)	37.0 (161)	55.3 (63)	0 < 1 < 2
For the patients with pain	Mean (SD)	Mean (SD)	Mean (SD)	
Worst pain intensity score	5.6 (2.5)	6.3 (2.5)	7.2 (2.3)	F=16.02, *P* < .001 0 < 1 < 2
Mean pain interference score	2.2 (2.0)	3.7 (2.5)	5.2 (2.6)	F=91.26, *P* < .001 0 < 1 < 2

Abbreviations: NS = not significant, SD = standard deviation.

a Clinically meaningful cutoff scores.

**Table 2:** Differences in Subscale and Total Scores for the Depression, Sleep Disturbance, and Cognitive Function Measures Among the Joint Anxiety and Depression Profiles at Enrollment

Measures	Low Anxiety and Low Depression (0) 57.5% (n = 764)	Moderate Anxiety and Moderate Depression (1) 33.7% (n = 448)	High Anxiety and High Depression (2) 8.8% (n = 116)	Statistics
	Mean (SD)	Mean (SD)	Mean (SD)	

Center for Epidemiological Studies–Depression (CES-D) Scale				
Somatic symptoms subscale	3.9 (3.0)	7.6 (3.5)	12.1 (4.0)	F = 397.73, *P* < .001 0 < 1 < 2
Depressed affect subscale	1.4 (2.0)	5.1 (3.5)	12.3 (4.2)	F = 821.15, *P* < .001 0 < 1 < 2
Positive affect subscale	10.2 (2.3)	7.6 (2.5)	4.8 (2.5)	F = 338.67, *P* < .001 0 > 1 > 2
Interpersonal problems subscale	0.1 (0.5)	0.3 (0.7)	1.2 (1.4)	F = 126.90, *P* < .001 0 < 1 < 2
Total CES-D score (≥16)	7.3 (5.1)	17.4 (6.5)	32.9 (8.4)	F = 1104.41, *P* < .001 0 < 1 < 2
General Sleep Disturbance Scale (GSDS)				
Quality of sleep (≥3)	2.8 (1.7)	3.8 (1.6)	4.9 (1.5)	F = 104.02, *P* < .001 0 < 1 < 2
Quantity ofsleep (≥3)	4.6 (1.5)	4.6 (1.7)	5.0 (1.9)	F = 3.52, *P* = .030 0 < 2
Sleep onset latency (≥3)	2.0 (2.1)	3.1 (2.2)	4.5 (2.2)	F = 66.43, *P* < .001 0 < 1 < 2
Mid-sleep awakenings (≥3)	4.8 (2.3)	5.0 (2.1)	5.6 (1.9)	F = 7.73, *P* < .001 0 < 2
Early awakenings (≥3)	3.1 (2.5)	4.0 (2.3)	5.2 (2.1)	F = 48.63, *P* < .001 0 < 1 < 2
Medications for sleep (≥3)	0.5 (0.7)	0.7 (0.8)	1.0 (0.9)	F = 26.74, *P* < .001 0 < 1 < 2
Excessive daytime sleepiness (≥3)	2.1 (1.3)	3.2 (1.3)	4.1 (1.3)	F = 184.45, *P* < .001 0 < 1 < 2
Total GSDS Score (≥43)	45.2 (18.6)	59.2 (16.9)	74.5 (16.6)	F = 176.77, *P* < .001 0 < 1 < 2
Attentional Function Index				
Effective action subscale	7.0 (1.9)	5.2 (1.9)	4.1 (2.0)	F = 186.82, *P* < .001 0 > 1 > 2
Attentional lapses subscale	7.3 (1.9)	6.0 (1.9)	4.5 (1.8)	F = 135.96, *P* < .001 0 > 1 > 2
Interpersonal effectiveness subscale	7.5 (1.7)	6.1 (1.9)	4.7 (2.0)	F = 166.49, *P* < .001 0 > 1 > 2
Total AFI score(<5.0 = Low, 5 to 7.5 = Moderate, >7.5 = High)	7.2 (1.5)	5.6 (1.5)	4.4 (1.7)	F = 245.75, *P* < .001 0 > 1 > 2

Abbreviation: SD = standard deviation.

Numbers in parentheses indicate clinically meaningful cutoff scores.

## Layperson Summary

### What we investigated and why

Anxiety and depression are common symptoms in cancer patients. However, little is known about how these symptoms co-occur.

### How we did our research

Patients completed anxiety and depression questionnaires six times over two cycles of chemotherapy.

### What we have found

Over 40% of the patients receiving chemotherapy reported moderate to severe levels of both state anxiety and depression. Patients with the highest levels of state anxiety and depression had the poorest quality of life outcomes.

### What it means

Clinicians need to ask patients about these two symptoms and provide effective interventions.
