# Supplementary material for: Greater Symptom Burden and Poorer Quality of Life Outcomes Are Associated With The Co-Occurrence of Anxiety and Depression During Cancer Chemotherapy
Source: Semin Oncol Nurs. Author manuscript; Available in PMC 2026 May 9. (PMC13156918; doi:10.1016/j.soncn.2025.151809)
Supplement: MMC1 [file NIHMS2161421-supplement-MMC1.docx]

Supplementary Figure 1. Changes in State Anxiety ((Anx), left y-axis) and Depression ((Dep), right y-axis) scores over two cycles of chemotherapy for subgroups of patients with Low Anxiety and Low Depression (panel A), Moderate Anxiety and Moderate Depression (panel B), and High Anxiety and High Depression (panel C).
