# Supplementary material for: Greater Symptom Burden and Poorer Quality of Life Outcomes Are Associated With The Co-Occurrence of Anxiety and Depression During Cancer Chemotherapy
Source: Semin Oncol Nurs. Author manuscript; Available in PMC 2026 May 9. (PMC13156918; doi:10.1016/j.soncn.2025.151809)
Supplement: MMC3 [file NIHMS2161421-supplement-MMC3.docx]

Supplementary Table 1 – Differences in Demographic and Clinical Characteristics at Enrollment Among the Anxiety and Depression Latent Classes

| Characteristic | Low Anxiety and Low Depression (0)  57.5% (n=764) | Moderate Anxiety and Moderate Depression (1)  33.7% (n=448) | High Anxiety and High Depression (2)  8.8% (n=116) | Statistics |
| --- | --- | --- | --- | --- |
|  | Mean (SD) | Mean (SD) | Mean (SD) |  |
| Age (years) | 58.7 (11.9) | 55.4 (12.6) | 53.6 (12.5) | F = 16.00, p <.001  0 > 1 and 2 |
| Education (years) | 16.3 (3.0) | 16.1 (3.1) | 15.8 (3.1) | F = 1.90, p = .150 |
| Body mass index (kg/m^2^) | 26.0 (5.2) | 26.1 (6.1) | 27.3 (6.5) | F = 2.78, p = .062 |
| Alcohol Use Disorders Identification Test score | 2.9 (2.2) | 3.0 (2.6) | 3.4 (3.6) | F = 1.22, p = .295 |
| Karnofsky Performance Status score | 83.7 (11.6) | 76.1 (12.0) | 70.6 (10.9) | F = 96.36, p <.001  0 > 1 > 2 |
| Number of comorbid conditions | 2.2 (1.4) | 2.5 (1.4) | 3.3 (1.6) | F = 32.39, p <.001  0 < 1 < 2 |
| Self-administered Comorbidity Questionnaire score | 4.9 (2.9) | 5.8 (3.2) | 7.9 (4.0) | F = 50.76, p <.001  0 < 1 < 2 |
| Time since diagnosis (years) | 2.0 (3.6) | 2.1 (4.0) | 1.8 (4.7) | KW = 0.93, p = .628 |
| Time since diagnosis (median, years | 0.42 | 0.42 | 0.42 |  |
| Number of prior cancer treatments | 1.6 (1.5) | 1.6 (1.5) | 1.8 (1.6) | F= 1.02, p= .363 |
| Number of metastatic sites including lymph node involvement^a^ | 1.3 (1.2) | 1.3 (1.3) | 1.2 (1.2) | F = 0.06, p = .943 |
| Number of metastatic sites excluding lymph node involvement | 0.8 (1.0) | 0.8 (1.1) | 0.7 (1.1) | F = 0.18, p = .837 |
| MAX2 score | 0.17 (.08) | 0.18 (.08) | 0.18 (0.9) | F = 3.43, p = .033  no significant pairwise contrasts |
|  | % (n) | % (n) | % (n) |  |
| Gender (% female) | 74.6 (569) | 82.1 (368) | 83.6 (97) | Χ^2^ = 11.80, p = .003  0 < 1 |
| Self-reported ethnicity  White  Asian or Pacific Islander  Black  Hispanic, Mixed, or Other | 71.2 (537)  12.6 (95)  8.1 (61)  8.1 (61) | 69.1 (306)  12.2 (54)  5.9 (26)  12.9 (57) | 60.5 (69)  12.3 (14)  7.0 (8)  20.2 (23) | Χ^2^ = 19.82, p = .003  NS  NS  NS  0 < 1 and 2 |
| Married or partnered (% yes) | 69.4 (522) | 60.9 (270) | 44.7 (51) | Χ^2^ = 29.78, p <.001 0 > 1 > 2 |
| Lives alone (% yes) | 18.5 (139) | 24.6 (109) | 30.7 (35) | Χ^2^ = 12.32, p = .002  0 < 1 and 2 |
| Currently employed (% yes) | 40.8 (307) | 27.8 (124) | 24.1 (28) | Χ^2^ = 27.22, p=<.001  0 > 1 and 2 |
| Annual household income  Less than $30,000  $30,000 to $70,000  $70,000 to $100,000  Greater than $100,000 | 12.9 (87)  20.1 (136)  19.1 (129)  48.0 (325) | 21.7 (88)  23.9 (97)  15.3 (62)  39.2 (159) | 42.5 (45)  17.9 (19)  9.4 (10)  30.2 (32) | KW = 40.76, p <.001  0 > 1 > 2 |
| Childcare responsibilities (% yes) | 19.9 (149) | 25.0 (109) | 26.5 (30) | Χ^2^ = 5.59, p = .061 |
| Elder care responsibilities (% yes) | 6.6 (46) | 10.2 (41) | 7.6 (8) | Χ^2^ = 4.59, p = .101 |
| Past or current history of smoking (% yes) | 33.5 (753) | 35.7 (156) | 45.7 (53) | Χ^2^ = 6.62, p = .036  0 < 2 |
| Exercise on a regular basis (% yes) | 73.8 (555) | 67.4 (293) | 64.9 (72) | Χ^2^ = 7.68, p = .022  no significant pairwise contrasts |
| Specific comorbid conditions (% yes) | | | | |
| Heart disease | 5.8 (44) | 6.0 (27) | 3.4 (4) | Χ^2^ = 1.19, p = .551 |
| High blood pressure | 31.4 (240) | 27.2 (122) | 34.5 (40) | Χ^2^ = 3.41, p = .182 |
| Lung disease | 9.8 (75) | 12.1 (54) | 19.0 (22) | Χ^2^ = 8.68, p = .013  0 < 2 |
| Diabetes | 9.0 (69) | 8.3 (37) | 11.2 (13) | Χ^2^ = 0.99, p = .609 |
| Ulcer or stomach disease | 4.1 (31) | 5.1 (23) | 9.5 (11) | Χ^2^ = 6.45, p = .040  0 < 2 |
| Kidney disease | 0.9 (7) | 1.8 (8) | 3.4 (4) | Χ^2^ = 5.18, p = .075 |
| Liver disease | 6.7 (51) | 6.5 (29) | 5.2 (6) | Χ^2^ = 0.38, p = .829 |
| Anemia or blood disease | 10.6 (81) | 13.4 (60) | 19.0 (22) | Χ^2^ = 7.33, p = .026  0 < 2 |
| Depression | 8.8 (67) | 26.3 (118) | 60.3 (70) | Χ^2^ = 194.86, p <.001  0 < 1 < 2 |
| Osteoarthritis | 11.9 (91) | 11.4 (51) | 15.5 (18) | Χ^2^ = 1.52, p = .468 |
| Back pain | 19.9 (152) | 30.4 (136) | 45.7 (53) | Χ^2^ = 42.87, p <.001  0 < 1 < 2 |
| Rheumatoid arthritis | 3.0 (23) | 2.9 (13) | 5.2 (6) | Χ^2^ = 1.69, p = .430 |
| Cancer diagnosis  Breast cancer  Gastrointestinal cancer  Gynecological cancer  Lung cancer | 39.7 (303)  32.1 (245)  17.0 (130)  11.3 (86) | 39.3 (176)  29.9 (134)  18.5 (83)  12.3 (55) | 49.1 (57)  21.6 (25)  15.5 (18)  13.8 (16) | Χ^2^ = 7.45, p =.281 |
| Prior cancer treatment  No prior treatment  Only surgery, CTX, or RT  Surgery and CTX, or surgery and RT, or CTX and RT  Surgery and CTX and RT | 26.4 (196)  40.4 (300)  21.7 (161)  11.6 (86) | 23.9 (104)  44.5 (194)  17.4 (76)  14.2 (62) | 20.9 (24)  42.6 (49)  16.5 (19)  20.0 (23) | Χ^2^ = 11.73, p =.068 |
| Metastatic sites  No metastasis  Only lymph node metastasis  Only metastatic disease in other sites  Metastatic disease in lymph nodes and other sites | 31.9 (241)  21.4 (162)  22.5 (170)  24.2 (183) | 32.4 (143)  22.2 (98)  20.0 (88)  25.4 (112) | 35.7 (41)  24.3 (28)  17.4 (20)  22.6 (26) | Χ^2^ = 2.89, p= .823 |
| Receipt of targeted therapy  Only chemotherapy  Only targeted therapy  Both chemotherapy and targeted therapy | 68.4 (514)  3.3 (25)  28.2 (212) | 71.2 (311)  2.3 (10)  26.5 (116) | 75.2 (85)  3.5 (4)  21.2 (24) | Χ^2^ = 3.74, p = .442 |
| Cycle length  14-day cycle  21-day cycle  28-day cycle | 43.2 (329)  49.3 (375)  7.5 (57) | 41.0 (181)  51.5 (227)  7.5 (33) | 35.1 (40)  59.6 (68)  5.3 (6) | KW = 1.63, p = .444 |
| Emetogenicity of the CTX regimen  Minimal/low  Moderate  High | 18.9 (144)  63.2 (481)  17.9 (136) | 19.7 (87)  58.4 (258)  21.9 (97) | 23.7 (27)  57.0 (65)  19.3 (22) | KW = 1.34, p = .512 |
| Antiemetic regimen  None  Steroid alone or serotonin receptor antagonist alone  Serotonin receptor antagonist and steroid  NK-1 receptor antagonist and two other antiemetics | 7.9 (59)  20.4 (152)  49.6 (370)  22.1 (165) | 6.3 (27)  20.3 (87)  46.9 (201)  26.6 (114) | 5.4 (6)  23.4 (26)  36.9 (41)  34.2 (38) | Χ^2^ = 12.03, p=.061 |

^a^Total number of metastatic sites evaluated was 9.

Abbreviations: CTX = chemotherapy, kg = kilograms, KW = Kruskal Wallis, m^2^ = meters squared, n/a = not applicable, NK-1 = neurokinin-1, NS = not significant, RT = radiation therapy, SD = standard deviation
