# Supplementary figures and images for: Greater Symptom Burden and Poorer Quality of Life Outcomes Are Associated With The Co-Occurrence of Anxiety and Depression During Cancer Chemotherapy
Source: Semin Oncol Nurs. Author manuscript; Available in PMC 2026 May 9. (PMC13156918; doi:10.1016/j.soncn.2025.151809)

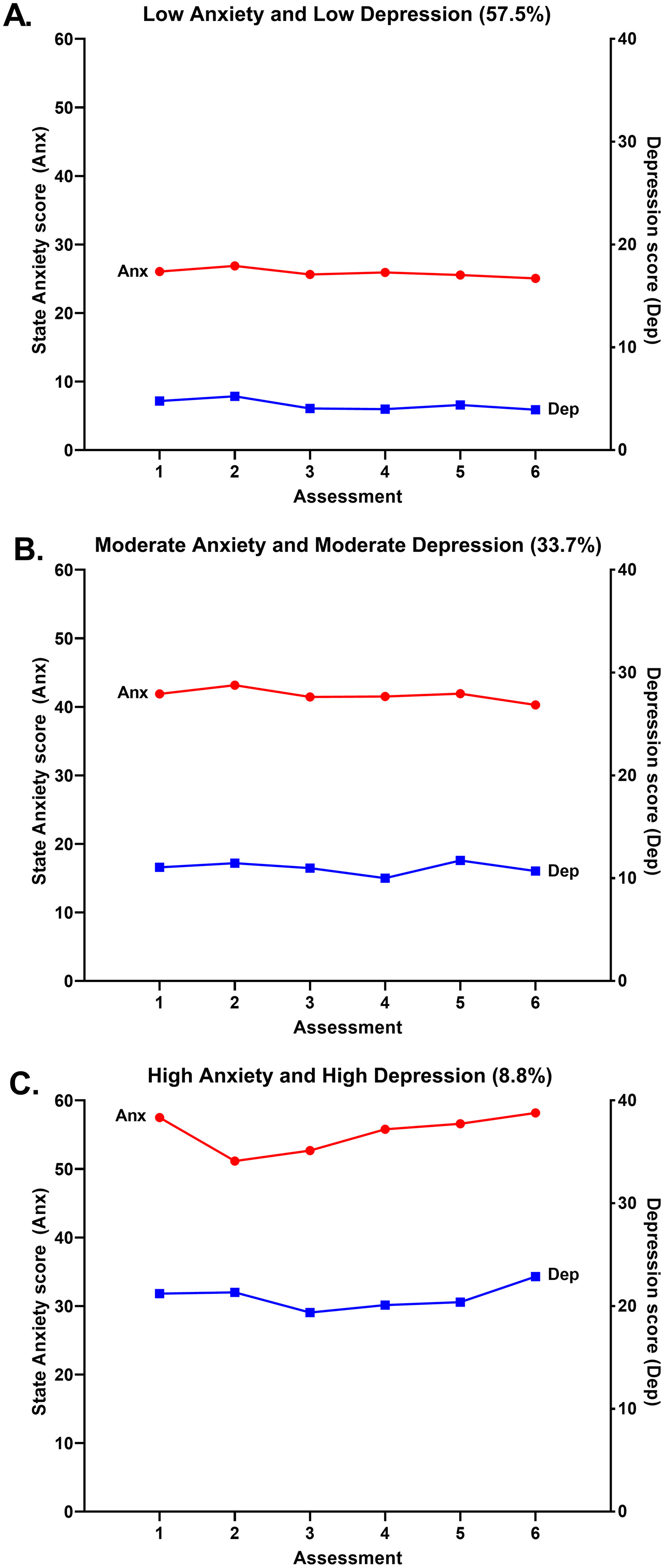

Supplement: MMC2 [file NIHMS2161421-supplement-MMC2.jpg]
